# Armed Oncolytic Myxoma Virus Induces Systemic Antitumor Immunity Against Solid Tumors in Immunocompetent Mice

**DOI:** 10.21203/rs.3.rs-10093553/v1

**Published:** 2026-07-09

**Authors:** Jacqueline Carmona, Junior A. Enow, Deon Nguyen, Mackenzie Cashen, Ami D. Gutierrez-Jensen, Natalie Reed, Manuel C. Marquez, Alexandra Lucas, Grant McFadden, Masmudur M. Rahman

**Affiliations:** Biodesign Institute; Arizona State University; Arizona State University; Biodesign Institute; Biodesign Institute; Biodesign Institute; Biodesign Institute; Biodesign Institute; Arizona State University; Arizona State University

**Keywords:** Myxoma virus, armed oncolytic virus, colorectal cancer, IL-15, LIGHT, CT26 cells

## Abstract

Oncolytic viruses expressing immunostimulatory transgenes can enhance immune cells recruitment into the tumor bed and activate potent antitumor immune responses. In this study, we evaluated oncolytic activity of recombinant myxoma virus (MYXV) variants expressing murine LIGHT, murine mIL-15, or IL-15Rα-IL-15 fusion protein. All recombinant MYXVs demonstrated similar replication kinetics and cytotoxic activity in cancer cell lines. Their therapeutic efficacy was further assessed in a bilateral tumor model in immunocompetent mice, where only one tumor received intratumoral treatment. We observed significant tumor regression in both injected and uninjected contralateral tumors following treatment with MYXV expressing IL-15Rα-IL-15 (vMyx-IL15Rα) or mLIGHT (vMyx-mLIGHT), indicating the activation of systemic antitumor immunity. Additionally, vMyx-IL15Rα- treated mice survived significantly longer than any of the other treatments. Analysis of tumor-infiltrating lymphocytes revealed that vMyx-IL15Rα increased effector memory CD8^+^ T cells, natural killer (NK) cells, and NK-T cells, whereas vMyx-mLIGHT enhanced infiltration of effector memory CD4^+^ T cells and dendritic cells. Furthermore, serum cytokine profiling showed increased levels of type I antitumor cytokines and reduced levels of protumor inflammatory cytokines and chemokines. These findings demonstrate that arming MYXV with immunostimulatory cytokines enhances both local and systemic antitumor activity by remodeling the tumor microenvironment and promoting effective immune responses.

## Introduction

Oncolytic viruses (OVs) are promising cancer therapies because they selectively infect and kill cancer cells while leaving normal cells unharmed. Following infection of the cancer cells, OVs activate antiviral immunity as well as immunogenic cell death (ICD) that releases tumor-associated antigens and amplifies or reactivates an immunogenic anti-tumor response^1–6^. OVs have emerged as a versatile mode of immunotherapy against cancers, where genetic engineering allows for the insertion of transgenes to express cytokines and chemokines that activate immune responses to both viral and tumor antigens^7,8^. Both preclinical and clinical studies show that OVs armed with transgenes are frequently superior to their unmodified OV counterparts in activating strong anti-tumor innate and adaptive immune responses. For example, Talimogene Laherparepvec (T-VEC) was the first OV to be approved by the FDA for the treatment of advanced inoperable melanoma. T-VEC is an oncolytic herpes simplex virus (HSV) engineered to express granulocyte-macrophage colony-stimulating factor and lacks two immunomodulatory viral genes that contribute to pathogenesis during normal HSV-1 infection^9^. Many OVs currently in clinical trials are armed to express different anti-tumor cytokines such as TNF, IL-2, IL-7, IL-12, IL-15, IFN-β, and OX40 ligand^7,8^.

OV-mediated expression of IL-15 and its derivatives has shown promising results among the many immune-stimulating interleukins. IL-15 is a pleiotropic cytokine that plays an essential role in the maturation, activation, and proliferation of lymphocytes such as T, NK, and NK-T cells^10^. The main signaling mode for IL-15 is trans-presentation, where the IL-15 ligand interacts with the IL15 receptor alpha subunit (IL-15Rα) inside the cell and becomes bioactive upon reaching the cell surface^11^. The IL-15/IL-15Rα complex is trans-presented to IL15Rβ/γ-expressing cells like NK and CD8^+^ T cells, promoting their activation^12^. However, the IL-15 ligand has a short half-life (< 1hr in mice) that limits its bioactivity in the peripheral circulation and within the tumor bed. Fusion of the IL-15 ligand with the IL-15 binding domain of the IL-15Rα, known as the sushi domain, improves the bioavailability and bioactivity of IL-15 in blood circulation^13–15^. The FDA (Food and Drug Administration) approved IL15 superagonist, N-803 (ANKTIVA), as an immunotherapy for the treatment of bladder cancer^16^.

LIGHT is another innate and adaptive immune response activator (TNF superfamily member 14: TNFSF14) that is expressed as both a membrane-bound and secreted ligand. LIGHT was initially discovered as an immune ligand that shares the receptor for herpesvirus infection and that stimulates antiviral T-cell proliferation^17^. LIGHT has shown potent antitumor effects when delivered locally into solid tumors by stimulating tumor-specific memory T-cell responses and activating tumor cell apoptosis^18^. This dual function of LIGHT makes it a potent candidate for stimulating anti-tumor cellular responses during cancer therapy^18,19^.

Myxoma virus (MYXV), a leporipoxvirus, has been established as an OV for treating many diverse classes of cancers^20^. MYXV causes a mild local skin disease in the natural evolutionary host the South American rabbit (*Sylvilagus* sp.), but causes a lethal, systemic disease called myxomatosis in the European rabbits (*Oryctolagus cuniculus*)^21,22^. MYXV was also used as a biocontrol agent against feral European rabbits in Australia and Europe^23^. It was demonstrated that MYXV is nonpathogenic for all the non-rabbit animals tested, including mice and humans^24^. MYXV was developed as an oncolytic virus because of its unique tropism for cancer cells, where the intracellular antiviral defense pathways are compromised, allowing the virus to replicate and selectively kill cancer cells^20,25^. The oncolytic activity of MYXV has been tested using various preclinical cancer models, including Small Cell Lung Cancer, Ovarian Cancer, Glioblastoma, and Multiple Myeloma^20,26,27^. Although unmodified MYXVs have shown therapeutic effects in some models, transgene-armed oncolytic MYXVs can activate more effective antitumor immune responses and reduce tumor burden in immunocompetent animals^28–33^. Previous studies have shown that IL-15 armed MYXV has a limited effect on slowing tumor progression in immunocompetent mice^34^. However, expression of mIL-15Rα/IL-15 fusion protein from MYXV (vMyx-mIL15Rα) enhanced infiltration of NK cells and CD8^+^ T cells in the subcutaneous B16-F10 tumor compared to the vMyx-mIL15 or control unarmed virus^35^. This treatment also provided survival benefits, suggesting that OV-mediated expression of mIL-15Rα/IL-15 in the tumor bed provides enhanced antitumor immune responses. MYXV armed with murine LIGHT (vMyx-mLIGHT) has also shown promising results when the virus is delivered to the tumor sites in the lung^30^. vMyx-mLIGHT, when delivered with carrier PBMCs (peripheral blood mononuclear cells), increased survival and reduced tumor burden in a lung metastatic osteosarcoma disease model^30^. These studies prompted us to compare these MYXV-expressed ligands (IL-15, mIL-15Rα/IL-15 fusion, and LIGHT) in a model of induced antitumor immunity.

Here, we have compared the three transgene-armed recombinant MYXVs in a syngeneic mouse model to test whether these OVs can induce abscopal immunity against established cancers. All the armed viruses replicated at similar levels to WT-MYXV in mouse and human colon cancer cell lines, and the proteins were all expressed properly from the transgenes. To evaluate their efficacy against solid tumors, we used the mouse CT26 colon carcinoma cell line to generate tumors on both hind flanks of BALB/c mice. The transgene-armed MYXVs were injected into one tumor, and the anti-tumor immune effects were measured on both tumors. Treatment with MYXVs expressing mIL-15Rα/IL-15 or mLIGHT reduced tumor burden on both flanks and prolonged the survival of mice. This anti-tumor response was likely mediated by an increase of tumor-infiltrating lymphocytes, specifically CD8^+^ and NKT cells for mIL-15Rα/IL-15 and CD4^+^T and dendritic cells for mLIGHT. We also observed a decrease in circulatory immunosuppressive cytokines and an increase in the level of antitumor cytokines and chemokines in the mice treated with armed MYXVs. These findings support the importance of using armed oncolytic MYXVs to improve immunosuppressive TME in solid tumors.

## Results

### Myxoma virus infection and replication are not affected by transgene expression

**Figure 1:**
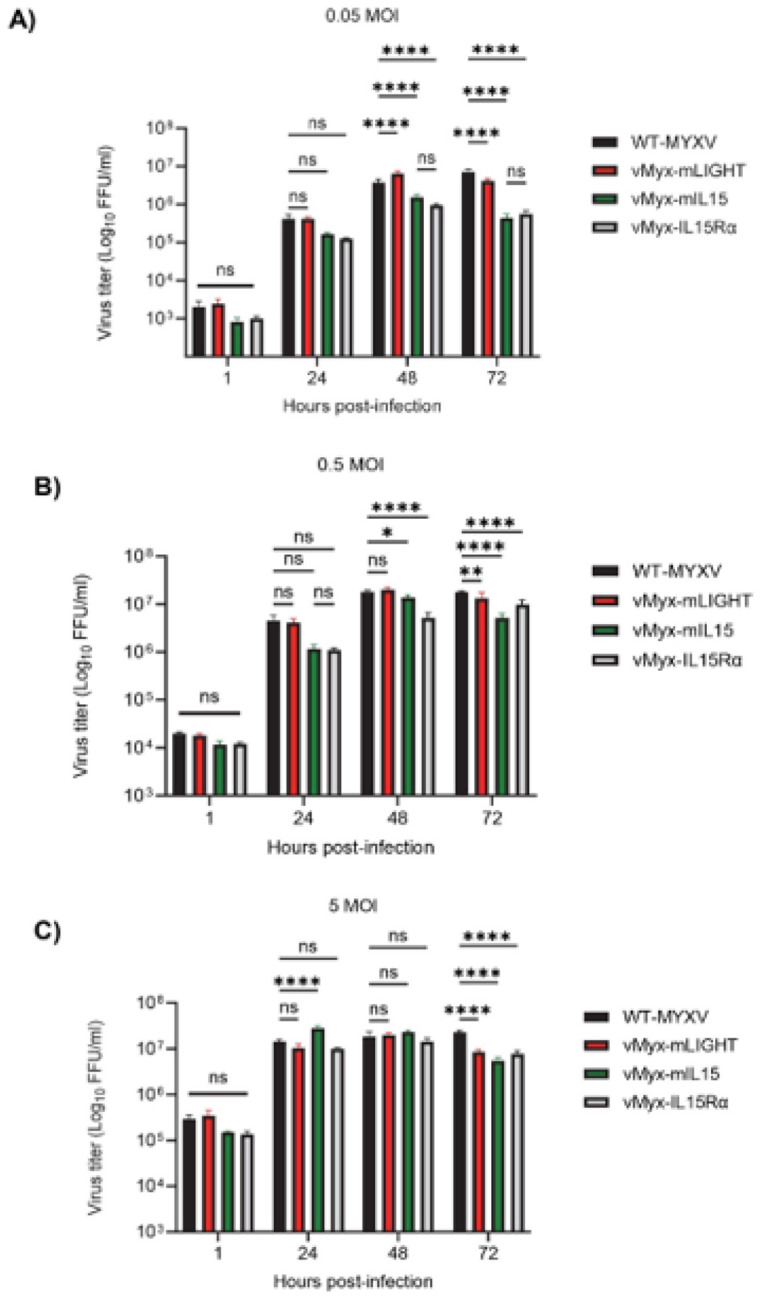
Transgene-armed oncolytic MYXVs exhibit a similar level of replication to WT-MYXV in CT26 cells. CT26 cells were infected with WT-MYXV, vMyx-mLIGHT, vMyx-mIL15, and vMyx- mIL15Rαviruses with three different MOI: A) 0.05, B) 0.5 and C) 5.0 for 1h, washed to remove unbound viruses, and added fresh complete media. Cells were harvested at indicated time points to determine progeny virus production by titration assays on permissive rabbit RK13 cells. Data represent ± SD and n = 3.

**Figure 2:**
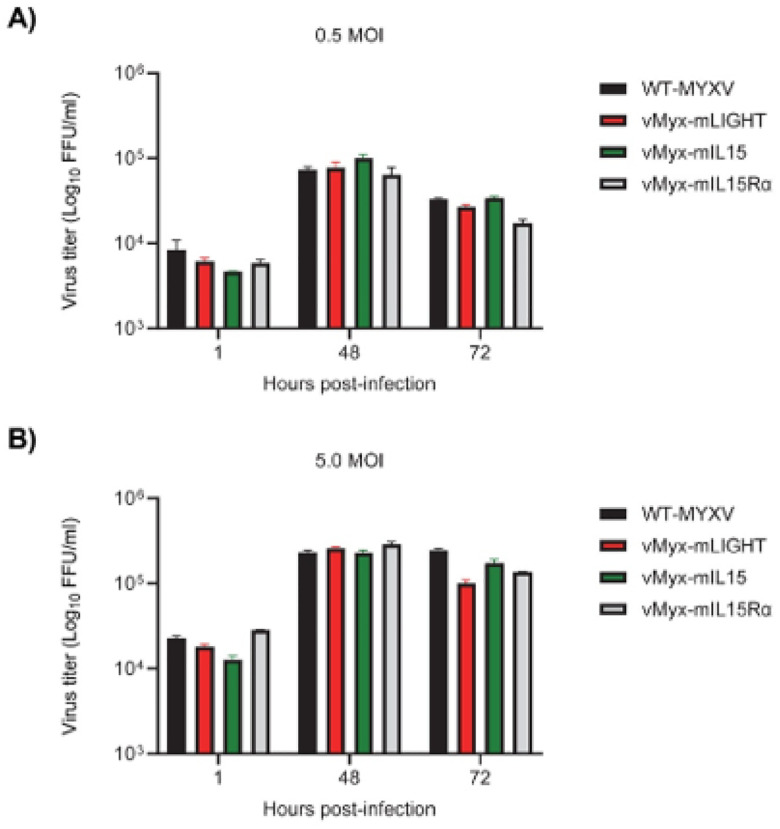
Transgene-armed oncolytic MYXVs exhibit a similar level of replication to WT-MYXV in the human colorectal carcinoma HCT116 cells. HCT116 cells were infected with WT-MYXV, vMyx-mLIGHT, vMyxmIL15, and vMyx-mIL15Rαviruses with two different MOI: A) 0.5 and B) 5.0 for 1h, washed to remove unbound viruses, and added fresh complete media. Cells were harvested at the indicated time points to determine progeny virus production by titration assays on permissive rabbit RK13 cells. Data represent ± SD and n = 3.

To test if expression of different transgenes from vMyx-mLIGHT, vMyx-mIL15, and vMyx-mIL15Rα has any effect on MYXV replication in cancer cells, we performed single-step (to reflect the number of infectious virus particles from initial infection and without cell-cell spread) and multi-step (to reflect the number of virus particles after cell-cell spread) virus replication assays using CT26 cells, a murine colorectal carcinoma cell line (Fig. 1). For this, the cells were infected with three different multiplicities of infection (MOI): 0.05 (Fig. 1A), 0.5 (Fig. 1B) or 5 (Fig. 1C) and virus titer was determined at 24-, 48- and 72-hour post infection (hpi). Like wild-type MYXV (WT-MYXV), all the transgene-armed MYXVs made progeny virions, as indicated by the strong increase in viral titers from 1hpi to 24hpi (Fig. 1). Maximum progeny virions were produced by all viruses at 48 hpi with different MOI, with a two to three log increase of virus titer from the initial inoculum (1hpi). These results suggest that in murine CT26 cells, expression of transgenes has no significant effect on virus replication compared to WT-MYXV. We tested replication of these viruses in HCT116 cells, a human colorectal carcinoma cell line. In HCT116 cells we observed about one log increase in titer for all the viruses at 48 hpi with an MOI 0.5 or 5 (Fig. 2A and 2B). Similar replication titers were also observed with WT-MYXV in other human colorectal cancer cell lines, such as HT29 and Colo205^36^. All the viruses tested had a reduction in viral titer at 72 hpi, possibly due to the activation of cell death. Overall, in both murine and human colorectal cancer cells, we observed no significant differences in the replication kinetics of these armed MYXVs when compared to WT-MYXV.

### Transgenic cytokines are expressed and secreted from the infected cells

**Figure 3:**
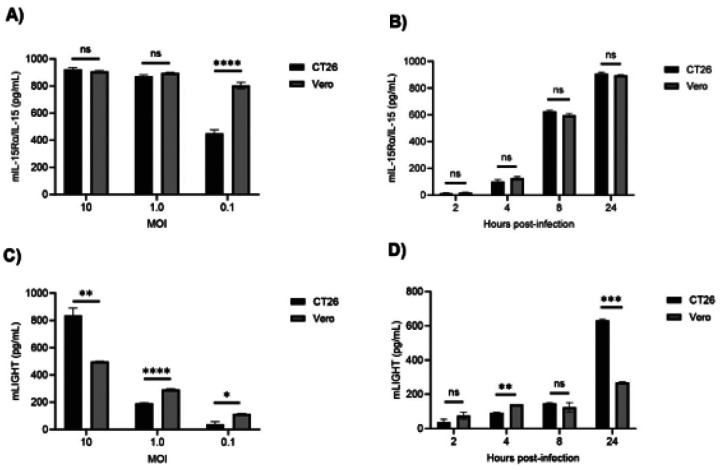
mIL15Rα/IL15 and mLIGHT are expressed and secreted from the virus-infected cells. CT26 or Vero cells in 6-well plates were infected with vMyx- mIL15Rα (A) or vMyx-mLIGHT (C) with the indicated MOIs, and media were collected at 24hpi for ELISA assays. In another set of experiments, cells were infected with vMyx- mIL15Rα (B) or vMyx-mLIGHT (D) with an MOI of 10, and media were collected at the indicated times post-infection for ELISA assays. Data represent ± SD and n = 3. Statistically significant differences are indicated. ^ns^ *P* > 0.05, * *P* < 0.05, ** *P* < 0.01, *** *P* < 0.001, **** *P* < 0.0001.

We tested the kinetics of expression of mLIGHT and mIL-15Rα/IL-15 proteins from two different cell types: Vero, a non-human primate cell line that completely supports MYXV replication, and CT26. We first infected these cells with vMyx-mIL15Rα or vMyx-mLIGHT with a MOI of 10, 1.0, and 0.1, collected the supernatant at 24 hpi, and measured the secreted protein levels using ELISA (Fig. 3A and 3C). When infected with vMyx-mIL15Rα, both CT26 and Vero cells secreted similar levels of mIL-15Rα/IL-15 at an MOI of 10 and 1, but Vero cells secreted more protein at the lower MOI of 0.1 (Fig. 3A). In contrast, the levels of mLIGHT protein were lower at MOI 0.1 and 1 and increased at MOI 10 for both CT26 and Vero cells (Fig. 3C). Since secretion of both proteins is robust at MOI 10, we tested the kinetics of secretion after infection by collecting the culture supernatants at different time points post infection (2, 4, 8 and 24 hpi). The mIL-15Rα/IL-15 complex was detected in both cell lines starting at 2 hpi and increased to more than 6-fold after 8 hours (Fig. 3B). The mLIGHT protein was detected in the supernatant at 2 hpi, followed by a 6-fold increase at 24 hpi in CT26 cells and a lower amount in Vero cells (Fig. 3D).

### Transgene-expressing MYXV constructs exhibit enhanced oncolytic activity

**Figure 4:**
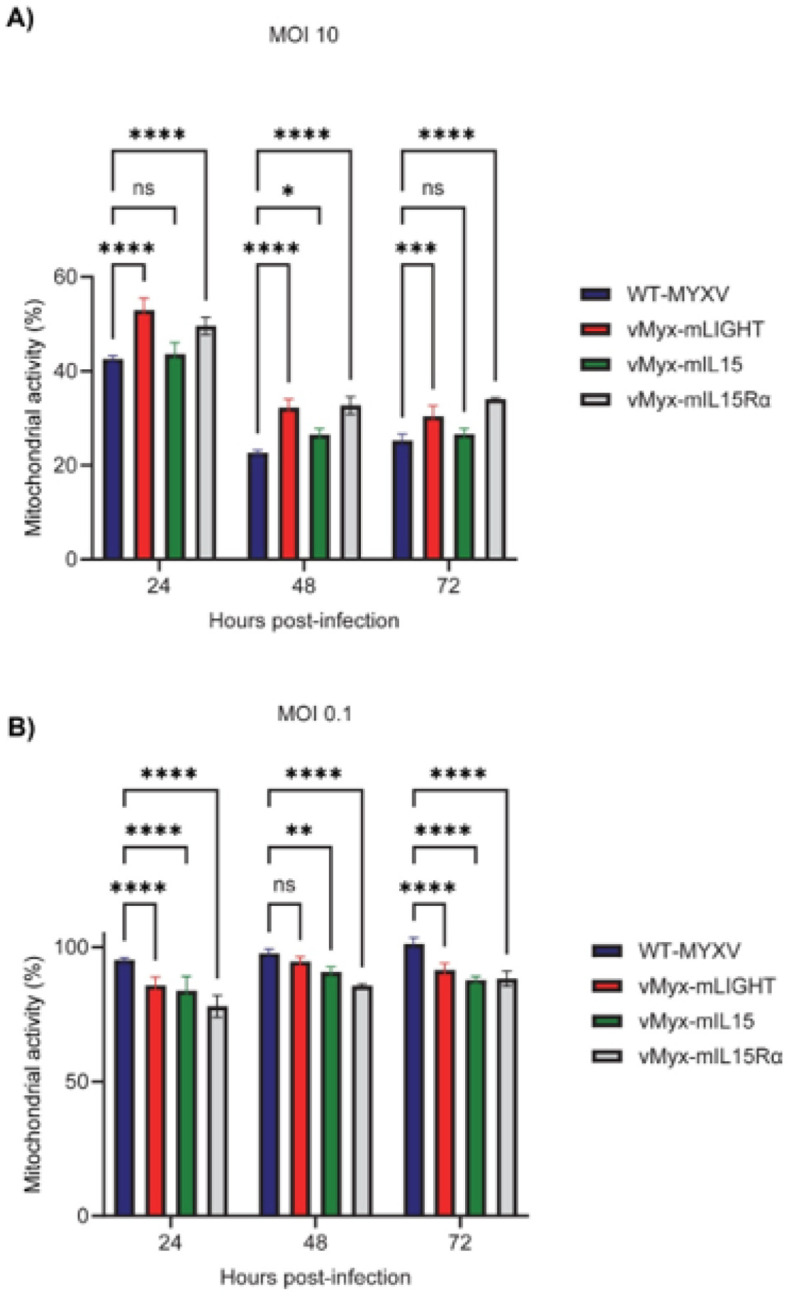
Transgene-armed oncolytic MYXVs significantly reduced the viability of CT26 cancer cells. CT26 cells in 96-well plates were left uninfected (mock) or infected with different oncolytic MYXVs with an MOI of 10 (A) or 0.1 (B) for 24, 48, and 72 hpi. Cell viability was measured using the MTS assay reagents. Data represent mean ± SD and n = 4. Statistically significant differences are indicated. ^ns^ *P* > 0.05, * *P* < 0.05, ** *P* < 0.01, *** *P* < 0.001, **** *P* < 0.0001.

To test if the different transgenes expressed by MYXV constructs can influence the oncolytic activity of MYXV, cell viability, as assessed by mitochondrial function, was measured using the MTS assay. CT26 cells were infected with two different MOIs, 10 and 0.1, and cell viability was measured at 24, 48, and 72 hpi (Fig. 4). Cell viability was significantly reduced at MOI 10 with WT-MYXV and all the transgeneexpressing MYXV constructs. Compared to the untreated control cells, between 40–60% cells were viable at 24 hpi, and between 20–40% cells were viable at 48 and 72 hpi (Fig. 4A). At an MOI of 0.1, cell viability was around 95% for WT-MYXV and significantly lower for all armed MYXVs, ranging from 75% (vMyx-mIL15Rα) to 85% (vMyx-mLIGHT), and it did not decrease further after 48 or 72 hpi. This is probably due to the proliferation of uninfected cells compared to the viral killing at a low MOI of infection (Fig. 4B). Taken together, these results suggest that with all the tested viruses, high MOI is needed to inhibit cell proliferation and that the armed MYXVs are better at tumor killing than the unarmed virus at a low MOI.

### MYXV construct expressing IL-15/IL-15Rα and mLIGHT shows superior tumor regression compared to WT and mIL-15 expressing viruses in the syngeneic mouse cancer model

**Figure 5:**
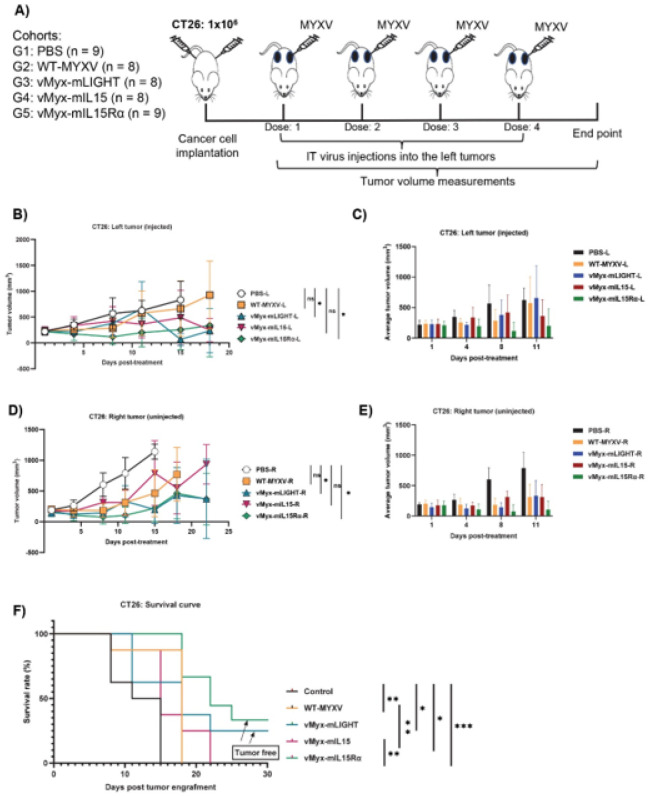
Transgene-armed oncolytic MYXVs reduced tumor burden and prolonged survival of immunocompetent mice. A) Diagram of experimental setup. BALB/c mice were inoculated with CT26 cells at day 0 via SQ injection on both flanks. Animals received four doses of intratumoral injections of different MYXV constructs on the left flank. B-E) Tumor volumes on the left and the right flanks were measured on different days after the start of treatments. Results are presented as mean ± SEM. F) Kaplan-Meier survival curves comparing animals with different treatment groups. ^ns^ *P* > 0.05, * *P* < 0.05, ** *P* < 0.01, *** *P* < 0.001, **** *P* < 0.0001.

To determine the therapeutic effect of the transgene expressing oncolytic MYXVs, CT26 cells were injected subcutaneously (SQ) in syngeneic BALB/c mice on both sides of the flank to generate tumors (Fig. 5A). After the tumor size reached ~ 50–100mm^3^ on both flanks, the animals were randomly assigned to different treatment groups such that each group maintained the same average tumor size. Mice (n = 8–9) received a total of four treatments within the first two weeks via intratumoral (IT) injection on the left flank tumor. The tumor burden on both sides of the flank was measured 2–3 times every week, and the mice were euthanized once the tumors reached ~ 1200–1500 mm^3^ in size on either side or due to any health concerns. Consistent with the previous reports^37^, CT26 tumors in the control PBS-treated mice grew rapidly, and all mice in this cohort reached criteria for euthanasia 15 days after the start of treatments (Fig. 5B, 5D, 5F). Treatment with WT-MYXV delayed tumor growth for both treated and contralateral flanks, the average tumor size increased by > 60% after day 15, and most mice in this cohort reached endpoint criteria 3 days later (Fig. 5B–5E, Suppl. Fig. S1A-1B, S1F-1G). In contrast, by the second treatment on day 4 with vMyx-mLIGHT or vMyx-mIL15Rα, the size of the injected and contralateral tumors was significantly smaller compared to PBS or WT-MYXV (Fig. 5C, 5E). The reduction in tumor growth rate continued 7 days after the last treatment, where six of the nine tumors injected with vMyx-mIL15Rα and two of the eight tumors injected with mLIGHT were regressed to a size that was not measurable (Suppl. Fig. S1C-1J). In total, two mice treated with vMyx-mLIGHT and two mice treated with vMyx-mIL15Rα were tumor-free 30 days after the initial treatment (Fig. 5F). During treatment, six out of eight tumors injected with vMyx-mIL15 were smaller than those injected with PBS or WT-MYXV, but unlike the mLIGHT or mIL15Rα/IL15 expressing viruses, vMyx-mIL15 failed to induce an abscopal effect after day 11, and the untreated side rapidly grew until all mice reached endpoint by day 22 (Suppl. Fig. S1D, S1I).

### Treatment with vMyx-mIL15Rα and vMyx-mLIGHT induces lymphocyte infiltration into the treated and untreated tumor beds

**Figure 6:**
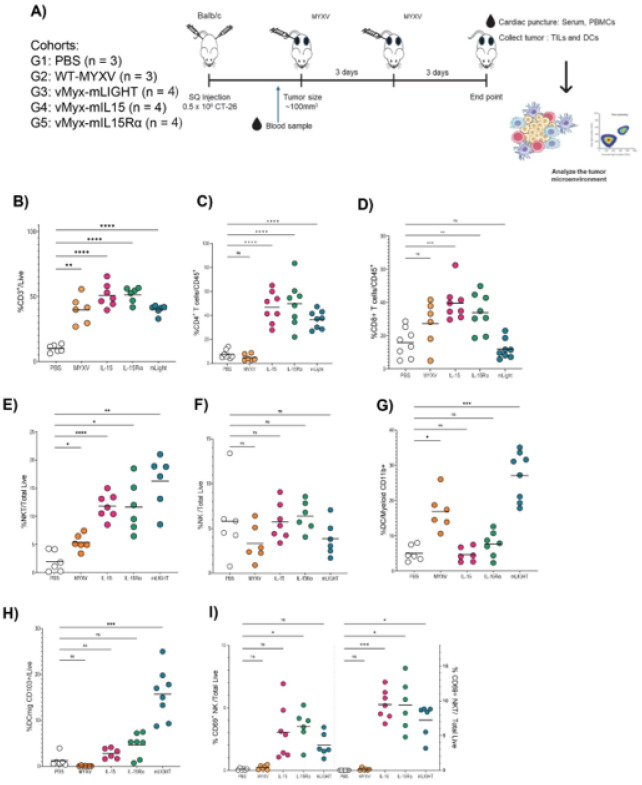
Treatment with transgene-armed oncolytic MYXVs enhanced infiltration of immune cells into the tumor microenvironment. A) Schematic diagram of BALB/c mice implanted subcutaneously with CT26 on both hind flanks and treated with different MYXVs as indicated. Tumors were processed to obtain single-cell suspensions for flow cytometric staining and analysis. Cells were divided into three staining panels to phenotype: B) CD3^+^ T cell lymphocytes, C) CD4^+^ T cells, D) CD8^+^ T cells, E) NKT cells, F) NK cells, G) DCs, H) CD103^+^ DCs, and I) Activated (CD69^+^) NKT and NK cells. The percentage number of cells for both tumors is presented in a scatter dot plot with group average. PBS: white circle; WT-MYXV: orange; mIL15: pink; mIL15Ra-IL15: green; mLIGHT: blue. Statistically significant differences were calculated by Brown-Forsythe and Welsh ANOVA, followed by Dunnett’s T3 multiple comparison test. * *P* < 0.03, ** *P* < 0.004, **** *P* < 0.0001.

To characterize changes in the phenotype and quantity of tumor-infiltrating lymphocytes, we first performed immunohistochemical (IHC) staining of tumor tissues from both virus-treated and untreated contralateral tumors. In this experiment, tumors were treated twice via IT delivery of WT-MYXV or armed MYXVs and were harvested 6 days after the first treatment (Suppl. Fig. S2A). Tumor sections of mice treated with vMyx-mIL15Rα showed a significant increase in the number of CD8^+^ T cells in both treated and untreated tumors compared to control, WT-MYXV, or vMyx-mLIGHT-treated mice (Suppl. Fig. S2B and S2C). Treatment with vMyx-mIL15 significantly enhanced CD8^+^ T cell recruitment on the injected side but not into the untreated contralateral tumors (Suppl, Fig. S2B and S2C). Based on the immunostimulatory properties of IL-15 and LIGHT, we performed multiparametric flow cytometry to further characterize and quantify lymphocyte infiltration into the tumor bed. Again, one of the tumors was treated twice (3 days apart) via IT injections with WT-MYXV or armed MYXVs, and both tumors were harvested 3 days after the second injection (Fig. 6A). Recruitment of CD3^+^ T cells into the tumor bed increased significantly after treatment with all the viruses, including WT-MYXV, vMyx-mIL-15, vMyx-mIL15Rα, and vMyx-mLIGHT, compared to PBS-treated control tumors (Fig. 6B, Suppl. Fig. S3A and S3B, Suppl. Table S1, S2).

We observed a 4-fold increase in CD4^+^ T cell recruitment over PBS and WT-MYXV when treated with any of the armed MYXVs (Fig. 6C, Suppl. Fig. S3C and S3D, Suppl. Table S1). CD8^+^ T cells were 2-fold higher for vMyx-mIL-15 and vMyx-mIL15Rα-treated mice compared to the controls (Fig. 6D, Suppl Fig. S3E and S3F, Suppl Table S1). For tumors treated with vMyx-mIL-15 and vMyx-mIL15Rα, the ratio of CD4 + to CD8 + T cells is about 3 to 1, in contrast to vMyx-mLIGHT treatment, where CD4^+^ T cells outnumbered CD8^+^ T cells 6 to 1 (Suppl. Table S4). For all treatment groups, most of the CD4^+^ and CD8^+^ T cells recruited were effector memory cells (T_em_), CD4^+^ T cells had a higher proportion of T_em_ (> 90%) than CD8^+^ T cells (75–78%), and less than 2% of CD4^+^ T cells recruited were central memory (T_cm_) or naïve T cells (T_n_). Both CD4^+^ and CD8^+^ T_eff_ populations were significantly higher in all the armed MYXV-treated mice. CD4^+^ T_eff_ were 22-, 37- and 3.7-fold higher than PBS while CD8^+^ T_eff_ cells were 15.9-, 14.9- and 13.6-fold higher than PBS for tumors treated with vMyx-mIL-15, vMyx-mIL15Rα, and vMyx-mLIGHT, respectively (Supp. Table S1–4).

The presence of Natural Killer T cells (NKT: CD3^+^NK1.1^+^) was higher in all armed MYXVs-treated tumors with an increase of more than 3-fold over PBS or WT-MYXV treatment (Fig. 6E, Suppl Fig. S4A-4B), whereas the presence of Natural Killer cells (NK: CD3^−^NKp46^+^) did not increase with any of the armed MYXVs when compared to control or WT-MYXV treatment (Fig. 6F, Suppl. Table S2). However, the percentage of activated NKT and NK cells was significantly higher for all tumors treated with the armed viruses, specifically mIL-15 and mIL-15Rα/IL-15 expressing MYXVs, where 10% of NKT and 3% of NK cells showed markers of activation (Fig. 6I). Since mLIGHT is known to recruit antigen-presenting cells into the tumor bed, we probed for dendritic cells (DCs: CD11b^+^CD11c^+^MHCII^+^) and found that treatment with vMyx-mLIGHT significantly increased the levels of DCs 5-fold over PBS (Fig. 6G–H, Suppl. Fig. S4C4D, Suppl. Table S3). About 5% of DCs in the control group were migratory DCs (CD11b^+^, CD11c^+^, MHCII^+^, CD103^+^), but treatment with mIL-15-, mIL15Rα/IL15- and mLIGHT-armed viruses increased their number by 2, 3, and 13-fold, respectively (Fig. 6G–H, Suppl. Fig. S4C-4D, Suppl. Table S3). These results indicate that transgene-armed oncolytic MYXVs recruit antitumor immune cells in both the injected and distal TME.

### Treatment with armed MYXVs induces unique signatures of cytokine upregulation

**Figure 7:**
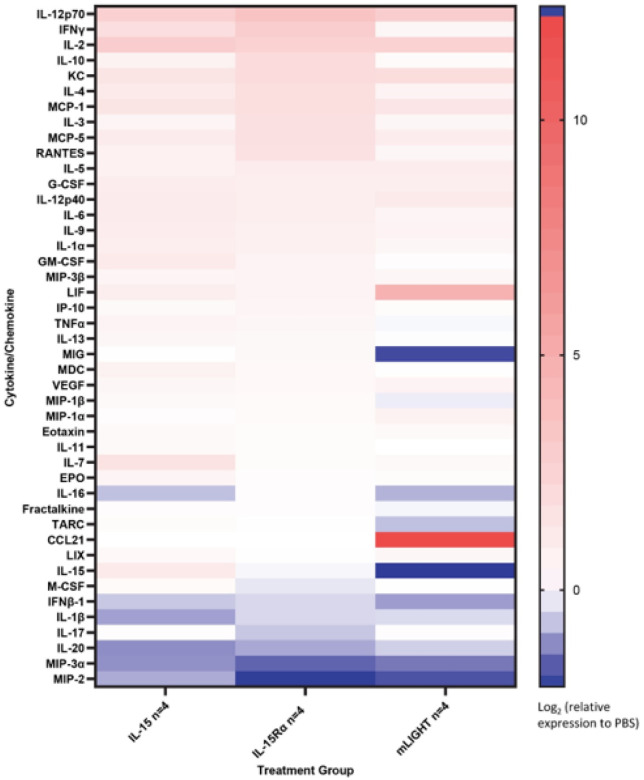
Treatment with transgene-armed oncolytic MYXVs induces changes in the level of circulatory cytokines. Serum was isolated from blood collected via cardiac puncture 3 days after the second intratumoral virus treatment. Antibody microarray of 44 cytokines was measured in duplicate per sample per cytokine. Raw fluorescent data were log converted and analyzed using a multiple linear regression model to find the concentration (pg/mL). The heatmap shows the log_2_ of the relative expression over the PBS control.

Cancer progression is a dynamic process that involves tumor and non-transformed cells interacting with cells of the immune system through signaling molecules like cytokines and chemokines. Since we observed a systemic anti-tumor effect after treatment with vMyx-mIL15Rα and vMyx-mLIGHT, we hypothesize that the increase in immune cell infiltration into the TME was accompanied by local and systemic changes in the circulatory levels of tumor-associated inflammatory cytokines and chemokines. To survey the differences in circulating cytokine and chemokine levels after treatment with armed MYXVs, we collected serum via cardiac puncture of mice used in the TME experiment above. We measured the level of 44 different circulating cytokines and chemokines using a multiplex ELISA microarray analysis. We found that treatment with mIL-15, mIL-15Rα/IL-15, and mLIGHT expressing viruses upregulated the expression of several proteins by more than 2-fold, among them are GM-CSF, IL-1α, IL-2, IL-4, IL-5, IL-12p70/IL-12p40, MCP-1, and MCP-5 and IFNγ (Fig. 7, Suppl. Fig S5, Suppl. Table S5). IFNγ, IL-12, and TNFα are part of the canonical Type I inflammatory antitumor response, while IL-1α, IL-4, and IL-5 can be anti- or pro-tumor in a context-dependent manner. On the other hand, 5 cytokines (IL-1β, IL-17, IL-16, IL-20, and IFNβ−1) and 2 chemokines (MIP-2, MIP-3α) known to be pro-tumor were downregulated after treatment with the armed MYXVs. These results indicate that transgene expression in the tumor bed alters the circulating cytokine levels in favor of antitumor immune responses.

## Discussion

Oncolytic poxviruses such as MYXV and vaccinia virus infect diverse cancer cell types irrespective of their tissue origin, making them suitable for oncolysis of heterogeneous cell types in the tumor bed^38,39^. These viruses have large dsDNA genomes, allowing the insertion of single or multiple therapeutic transgenes, such as cytokines and chemokines, that can activate potent antitumor immune responses. OV-mediated expression of therapeutic proteins in the tumor bed has shown multiple benefits in several preclinical and clinical studies compared with delivering the proteins alone^7,8,38^. Thus, engineered transgene-armed OVs are not only responsible for killing and lysis of cancer cells but also for activating higher levels of antitumor innate and adaptive immune responses. These stronger antitumor immune responses are significant in eliminating distal untreated or metastatic tumors and improving long-term survival. Here, we compared the oncolytic activities of the WT and three different transgene-armed oncolytic MYXVs using cell-based assays and a preclinical cancer model. Specifically, we used a syngeneic murine model to assess the local and abscopal antitumor effects of transgene-armed oncolytic MYXVs.

Transgene armed oncolytic MYXVs vMyx-IL15, vMyx-mIL15Rα, and vMyx-mLIGHT have been individually tested in different cancer models, showing increased survival and a reduced tumor burden compared to unarmed WT-MYXV^28–30,35,40^. However, their therapeutic efficacy, modulation of TME, and abscopal antitumor effects were not compared in the same preclinical cancer model. First, we evaluated the replication of the transgene-armed MYXVs in mouse and human colorectal carcinoma cell lines. Like the WT-MYXV, the armed MYXVs replicated and produced comparable levels of progeny, suggesting that transgene expression has no significant effect on virus replication. We further confirmed the expression and secretion of the transgenes from infected cells using cytokine-specific ELISA. Expression and secretion of murine IL-15 and IL-15Rα/IL-15 were previously confirmed in these constructs^35^. Here, we show that mLIGHT is also secreted from infected cancer cells at a much lower level than IL-15Rα-/IL-15; this is expected because LIGHT is first expressed as a cell-surface ligand that is then cleaved to be released as the soluble form of the ligand^41,42^. Since OVs reduce cancer cell viability using different mechanisms, we further tested whether transgene expression can alter the OV-mediated cell-killing effect. Using cell viability assay, we observed a comparable reduction in cell viability with high MOI (MOI 10) of infection with all the tested viruses. Interestingly, with low MOI (MOI 0.1), we observed significantly reduced cell viability with transgene-armed viruses than unarmed MYXV, possibly due to the anti-cancer cell-proliferation effect of IL-15 and LIGHT^18,43^.

To better understand the anticancer and immunostimulatory functions of these transgene-armed viruses, we used the CT26 syngeneic mouse model known for its consistent tumor engraftment and moderate levels of immune infiltration^37^. We compared the tumor burden, survival, and immune cell infiltration within the TME after intratumoral injections of PBS, WT- or armed-MYXVs into one of two flank tumors. In contrast to the untreated group (PBS-control) that quickly reached the endpoint within 12–15 days, mice treated with WT-MYXV survived only three days longer, reaching the endpoint by day 18. The delay of tumor growth could be explained by a significantly higher number of CD3^+^ T lymphocytes, specifically CD8^+^ T and NKT cells, compared to the PBS group (Fig. 6).

Mice treated with mIL-15-expressing MYXV showed a significant decrease in tumor size on the treated side but had no significant reduction of tumor burden (after last treatment) on the uninjected side and no significant extension of survival. This is not unexpected based on the short half-life of IL-15^44,45^. In contrast, mice treated with vMyx-mIL15Rα and vMyx-mLIGHT had a significant decrease in tumor burden by day 15 in both untreated and treated flanks, with a prolonged abscopal effect 10 days after the last treatment. Both groups had two tumor-free mice by day 40, but only vMyx-mIL15Rα significantly slowed tumor growth during and after treatment, thereby extending overall survival. The antitumor effect observed during and after treatment with mIL15 and mIL-15Rα/IL-15 is likely mediated by CD3^+^ T lymphocytes, specifically CD4^+^ T_em_, CD8^+^ T_em,_ and NKT cells, which were significantly higher on both the treated and untreated sides. Effector T cells (T_eff_) are likely involved in this antitumor response, since more than 10% and 22% of cells recruited into the TME were CD4^+^ T and CD8^+^ T_eff_ cells, respectively, compared to less than 1% for the PBS-treated group. Together, these results suggest that after 2 treatments with either mIL15 or the mIL-15Rα/IL-15-armed MYXVs, the anti-tumor response is likely mediated by both T_em_ and T_eff_ cells. The presence of T_eff_ in the TME is associated with better prognosis for solid tumors and blood malignancies^46,47^, but further study is required to characterize the type of effector cells recruited after treatment with armed-MYXVs and whether this population is short-lived or can differentiate into tumor-specific T_mem_ cells. Although the vast majority of CD4^+^ and CD8^+^ T cells in the PBS control group were effector memory cells, it is possible that these cells may have an exhausted phenotype that failed to control tumor growth or extend the survival of mice in this group. Investigation into the activation and exhaustion status of T_em_ cells in PBS-treated tumors is warranted and will likely underscore the use of OVs armed with immune-activating cytokines like mIL-15Rα/IL-15.

The mIL-15Rα/IL-15 complex can enhance proliferation and cytotoxic activity of NK and CD8^+^ T cells against cancer cells^48,49^, increase NK infiltration and their antitumor activity in preclinical murine tumor models^35,50,51^. Although we did not observe a significant increase in NK cells in the tumor bed with flow cytometry, the percentages after mIL-15Rα/IL-15 treatment were 2.3-fold higher than the control, and 9-fold higher on the treated side. Of importance, we found that the percentage of activated NK and NKT cells was significantly higher in the tumors treated with mIL-15Rα/IL-15 and mLIGHT-armed MYXVs. Specifically, after treatment with mIL-15Rα/IL-15 the number of activated NK and NKT cells (CD69^+^) increased from less than 0.1% in the PBS control group to about 3.4% and 9%, respectively. For tumors treated with mLIGHT, the percentage of activated NK and NKT cells increased to 2% and 7%, respectively, which indicates that the presence of both cytokines improved the activation status of immune cells. vMyx-mLIGHT has shown promising results in pancreatic cancer and lung metastatic osteosarcoma models^29,30^. Here, we show that treatment with mLIGHT can decrease tumor burden and enhance survival by rapidly changing the TME. After two treatments, we found a significant increase in CD8^+^ T_eff_ cells, from 1.5% in the PBS control to almost 21% in both treated and untreated sides. Most of the immune cells recruited into the tumor bed are CD4^+^ T_em_ cells, DCs, NKT cells, and activated NK cells. This suggests that the observed tumor burden regression and improved survival is initiated by the recruitment of antigen presenting cells and activation of anti-tumor NKT and NK cells. vMyx-mLIGHT slowed tumor growth and enhanced survival as evidenced by the progression free survival of two mice 40 days after the last treatment. Although we did not survey immune infiltration after more than 9 days or more than 2 treatments, it is likely that the regression in tumor size observed in the survival study was due to an expansion of CD8^+^ anti-tumor T cells followed by the initial expansion of antigen presenting cells documented by flow cytometry. Like mIL15Rα/IL15, vMyx-mLIGHT also had an abscopal effect with regression in both untreated and treated sides, accompanied by an increase in CD4^+^ T cells, DCs and activated NKT and NK cells.

IL-15 and LIGHT play important roles in both activation of anti-tumor responses and remodeling of the TME^18,52^. Primary signaling by IL-15 or LIGHT can downregulate or upregulate expression and signaling mediated by other cytokines and chemokines^53,54^. Therefore, we hypothesized that expression of mIL15, mIL15Rα/IL15, and mLIGHT within the TME via armed MYXVs would affect the level of other signaling molecules involved in inflammatory responses to tumor development. To better understand these changes in the signaling molecules, we assessed the levels of 44 different circulating cytokines and chemokines using a multiplex ELISA microarray analysis. We found that treatment with vMyx-mIL15, vMyx-mIL15Rα, and vMyx-mLIGHT upregulated the expression of 9 different proteins by more than 2-fold, among them include G-CSF, IL-2, IL-5, IL-12p40/p70, MCP-1 and MCP-5 (Suppl. Table 5, Fig. 7–8). This group of cytokines and chemokines have anti- and pro- tumor roles that are context dependent. For example, IL-12 cytokines like IL-12p70 have a pleiotropic effect on immune cells of the TME. They exert a strong antitumor response both in cell culture and in animals by polarization of T cells into a type 1 helper (T_h1_ effector phenotype), facilitate effector memory T cell generation and inhibit pathways that lead to T_h2_ and T_reg_ commitment^55,56^. Treatment with vMyx-mIL15 and vMyx-mIL15Rα increased the levels of IFNγ, another Type 1 cytokine involved in antitumor responses^57^. Other cytokines like IL-4 and IL-5 mediate Type 2 immune responses that can be anti-tumorigenic when they promote tissue regeneration and wound healing through the recruitment of macrophages and the remodeling of blood vessels^58^. However, their effect can also be pro-oncogenic when present during chronic inflammation^59^.

The antitumor effects of vMyx-mIL15, vMyx-mIL15Rα, and vMyx-mLIGHT treatments can in part be explained by lower serum levels of protumor cytokines and chemokines such as IL-1β, IL-17, MIP-2, IL-20, and MIP-3α. For example, IL-1β promotes the formation of pre-malignant lesions by activation of NF-kB signaling pathways and the creation of reactive oxygen species, leading to cell proliferation and evasion of apoptosis^60^. Another cytokine downregulated by armed MYXV treatment was IL-17A, which promotes tumorigenesis by decreasing CD8 + T cell activation, while encouraging neutrophil and myeloid-derived suppressor cell recruitment^61^. Higher levels of IL-17A have been linked to lower proliferation and activation of NK cells and impaired antitumor and antiviral responses due to IL-17A-mediated dampening response to IL-15 signaling mediated by the activation of Suppressor of Cytokine Signaling^62^. To end, we found lower serum levels of IL-20 and two different macrophage inflammatory proteins (MIP-2 and 3α), all of which have a documented protumor effect. IL-20 is a cytokine known to promote tumor formation by cell proliferation and evasion of apoptosis in lung, breast and ovarian cancer cells, while in animal models the use of anti-IL-20 antibody can slow tumor growth and shift M2-polarization in pancreatic ductal adenocarcinoma^63–65^. IL-20 can also remodel the TME by activating the JAK-STAT2-SOX2 signaling pathway that serves to suppress CD8^+^ and NK anti-tumor cell recruitment while increasing the number of myeloid-derived suppressor cells in an orthotopic breast cancer mouse model^66^. MIP-2 is a CXC chemokine also known as CXCL2 that can promote angiogenesis and tumor growth of CT26 tumors and can affect neutrophil recruitment and activation leading to chronic inflammation and dysfunction of the liver^67,68^. MIP-3α secretion is higher in patients with hepatocellular carcinoma, promotes metastasis by up-regulation of MMP9 in pancreatic adenocarcinoma and higher levels have been detected in colon, lung, pancreatic carcinomas and melanoma^69^. In summary, treatment with these armed MYXVs improved the TME by decreasing expression of pro-tumor inflammatory cytokines while promoting expression of anti-tumor cytokines and chemokines. However, further investigation is needed to clarify the changes in proteins like G-CSF. G-CSF level was over 2-fold higher in the serum of mice treated with armed-MYXVs, and it is known to promote protumor changes in the TME, like infiltration of M2 macrophages, myeloid-derived suppressor cells, and CD4^+^ T regulatory cells^70^.

The current study demonstrates that intratumoral delivery of armed-MYXVs can activate abscopal antitumor immune responses, mostly mediated by CD3^+^ T lymphocytes, specifically CD4^+^ T_em_ and CD8^+^ T_em_ and NKT cells, when treated with vMyx-mIL15 or vMyx-mIL15Rα and by DCs and NKT cells when treated with vMyx-mLIGHT. These results open the possibility of testing the mIL15Rα/IL15-armed oncolytic MYXV against metastatic cancers in combination with immune checkpoint inhibitors such as PD1/PD-L1 antibodies for immunologically cold tumors.

## Materials and methods

### Cells and viruses

RK13 (Cat #CCL-37), Vero (Cat #CCL-81), HCT-116 (Cat #CCL-247), and CT26 (Cat #CRL-2638) cells were purchased from the American Type Culture Collection (ATCC). Individual cell lines were tested for mycoplasma contamination monthly using a universal mycoplasma detection kit (Cat #30–1012K) from ATCC. RK13, Vero, and CT26 cells were cultured in Dulbecco’s Modified Eagle medium (DMEM; Cytiva) supplemented with 10% fetal bovine serum (FBS, Gibco), 2 mmol/L glutamine (Invitrogen), and 100 μg/ml of penicillin-streptomycin (P/S; Invitrogen). HCT116 cells were cultured in McCoy’s 5 (Cytiva) media supplemented with 10% FBS, 2 mmol/L glutamine, and 100 μg/ml of P/S. All the cultures were maintained at 37°C in a humidified 5% incubator. Individual cryovials were thawed and cells were grown for no more than 20 passages.

### Viruses and viral replication assay

vMyx-GFP, a WT-MYXV that expresses GFP under a poxvirus synthetic early/late (sE/L) promoter; vMyx-GFP-TdTomato, a WT-MYXV that expresses GFP under a poxvirus sE/L promoter and TdTomato under a poxvirus p11 late promoter; vMyx-mIL15, vMyx- mIL15Rα/IL15, and vMyx-mLIGHT were used in this study. The construction of these viruses was described before^29,35,71^. All the myxoma virus constructs were grown in RK13 or Vero cell lines. The viral stocks were prepared using sucrose cushion purification as described previously^72^.

Viral titers in different cancer cell lines were determined using a viral replication assay. The cells were seeded in 24 well plates (2 × 10^5^ cells/well). The next day, the cells were infected with the different MYXVs, and incubated for 1h at 37°C. After 1h, the unbound virus was washed away using DMEM, and fresh DMEM was added to the cells again. Cells were harvested at the indicated time points. Cells were harvested and stored in a −80 ° C freezer until needed or subjected to three freeze/thaw cycles followed by 1-minute sonication to release the viral particles from lysed cells. Afterward, the clarified virus was serially diluted in DMEM and added to RK13 cells, and after 48h, the GFP foci were counted using a fluorescent microscope. All assays and dilutions were performed in triplicate.

### Cell viability assay

To assess the viability of cancer cells after MYXV infection, CT26 cells were plated into a 96-well plate at a density of 1 × 10^4^ cells per well. The next day, cells were infected using different MOIs of the four different MYXV constructs. A minimum of four to five wells were used for each treatment condition, and untreated cells (mock) served as controls. Cell viability at 24h, 48h, and 72h post-infection was assessed using the MTS assay (CellTiter 96 Aqueous One Solution Cell Proliferation Assay; Promega Corporation) and a reader (VarioSkan Lux, Thermo Scientific) using the SkanIt Software v.4.1.

### ELISA assay for the detection of expressed proteins

The cells were seeded in 6- well plates at a density of 1 × 10^6^ cells/well and infected with the armed or unarmed MYXV constructs at MOI 0.1, 1 or 10 and incubated for 1h at 37°C. After 1h, the unbound virus was washed away using DMEM and replaced with complete DMEM media. Cell supernatants were collected at 2-, 4-, 8-, and 24-hours post-infection, centrifuged briefly to remove cellular debris, and collected into a new tube for storage at −80 ° C until needed for ELISA assays. The mouse IL-15/IL-15Rα complex ELISA kit (Invitrogen) and the mouse LIGHT/TNFSF14 DuoSet ELISA kit (R&D Systems) were used for the detection of mIL15Rα/IL15 and mLIGHT protein, respectively. All samples were prepared in triplicate, and the assays were done in duplicate according to the manufacturer’s instructions.

### Flow cytometry

To collect whole blood from BALB/c aged-matched male and female mice, we performed cardiac puncture 72 hours after the second treatment, and 400–800 μL of blood was collected into a 1.5 mL Eppendorf tube containing 1–3 units of heparin per 100 μL blood to prevent coagulation. After manual tube inversion, tubes were placed in ice and processed within 30 minutes. Briefly, the collected blood was washed with 10 mL PBS at room temperature (RT), centrifuged at 300 × *g* for 5 minutes, and lysed with 1 mL of RBC lysis buffer for every 100 μL of blood for 3 minutes at RT. Cells were centrifuged at 300 × *g* for 5 minutes at 4°C, the supernatant was discarded, and lysis was repeated 1 or 2 times more if needed. PBMCs were washed with 5–7 mL of PBS supplemented with 3% FBS (PBS + FBS) and resuspended in 1–2 mL of PBS + FBS to count cell density and viability using a hemocytometer and Trypan blue exclusion staining. PBMCs were incubated in a solution of FACS buffer (Mg^+ 2^/Ca^+ 2^-free PBS + 2% FBS + 0.1% Sodium Azide), Live/Dead Fixable ef780 viability dye, and FcRγ buffer (Miltenyi Biotec) at a 1:500 dilution for 20 min at 4°C. Then, PBMCs were separated into three staining groups and incubated for 30 minutes at 4°C with the specific stain mix for T, NK, and DC stains (see TIL section below for panel details), washed, and fixed with 1% paraformaldehyde solution for 10 minutes. Data were acquired with the Attune Nx T flow cytometer (Thermo Fisher Scientific) and analyzed with FlowJo (v10.10.0).

### Animal studies

All animal experiments were approved by the Institutional Animal Care and Use Committee (IACUC) of Arizona State University and conformed to all regulatory standards. Male and female BALB/C mice were purchased from the Jackson Laboratory at 6–8 weeks of age. After arrival, the animals were housed at the Biodesign Institute vivarium under the appropriate conditions. The animals were acclimatized for at least seven days before tumor implantation or any experimental procedures. All animal handling, housing, husbandry, and experimental protocols were performed according to the approved IACUC protocols and institutional standards. Cells (5 × 10^5^/mouse in 100 μL PBS) were subcutaneously injected into the flanks of BALB/C mice. When the average tumor volumes reached 50–100 mm^3^, the mice were randomized into different treatment groups. Each treatment group contained eight or nine animals, and virus injections were administered using 5 × 10^7^ FFU. Tumor size was measured three times a week with an electronic caliper (Fisher Sci. Traceable), and the tumor volume was calculated as: Volume = Min [length, width]^2^ × Max [length, width] × 0.5. Mice were monitored daily for signs of lethargy, weight loss, or pain. Once tumors reached 1.2–1.5 cm^3^, mice were euthanized by CO_2_ asphyxiation and cervical dislocation. For histology studies, the tumors were treated twice at 3-day intervals and were harvested three days after the last treatment.

### Euthanasia procedure

Mice were euthanized by using carbon dioxide (CO_2_) inhalation, followed by cervical dislocation, following AVMA Guidelines for the Euthanasia of Animals. The mice were placed in a clear cage, covered with the stainless-steel euthanasia lid, which was connected to a CO_2_ cylinder. Using the flow meter, a displacement rate of 30 to 70% of the chamber volume with CO_2_ per minute was used to control the flow of CO2. The animals were exposed to CO_2_ until the complete cessation of breathing was observed (5–10 minutes). Death was assured by cervical dislocation.

### Tumor-infiltrating lymphocyte isolation and flow cytometry immunophenotyping

Tumor samples were minced and digested for 2 hours at 37°C with constant stirring in a solution made of Liberase LT (50 μg/mL), DNase I (100 μg/mL), and 3% FBS in HBSS buffer. Once the digested tissue was filtered twice (70 um and 45 um), the cell suspension was washed once with PBS (3% FBS) and once with Mg^+ 2^/Ca^+ 2^-free PBS. Immune cells were enriched by histopaque-1083 centrifugation, pelleted, washed, and resuspended in PBS. Live cells were separated using the Dead Cell Removal kit and LS Columns (Miltenyi Biotech), washed three times with MACS buffer, and resuspended in ice-cold FACS buffer. Cells were counted and divided into three staining groups: T cell stain, NK cell stain, and DC stain. Before staining, cells were incubated with FcRγ buffer (1:500 FACS) and Live/Dead Fixable ef780 viability dye for 20 min at 4°C. After washing, cells were stained with the corresponding antibody panel for 30 min at 4°C, washed, and fixed in 1% PFA solution for 10 minutes and resuspended in PBS. Data were acquired with the Attune Nxt flow cytometer (Thermo Fisher Scientific) and analyzed with FlowJo (v10.10.0). T cell antibody panel: CD45 (BD-V450), CD3 (BV-650 Biolegend), CD8 (BV711 Biolegend), CD4 (FITC Biolegend), CD44 (PE Biolegend), and CD62L (APC Biolegend). NK antibody panel: CD45 (V450, BD), CD3 (BV711 Biolegend), NK1.1 (AF488 Biolegend), NKp46 (PE Biolegend), CD69 (APC Biolegend). DC antibody panel: CD45 (Bb700 Biolegend), CD11b (Bb515 Biolegend), CD11c (Pacific Blue BD), MHCii-A/B/C (BV605 Biolegend), CD103 (PE Biolegend).

### Histological analysis

Tumor tissues were formalin-fixed, paraffin-embedded, and sections were stained with standard hematoxylin and eosin (H&E) staining. After immunohistochemical (IHC) staining, three high-power fields were assessed for each specimen, and the positively stained cells per 40x field were counted. Sections were examined using an Olympus BX51 microscope with 4x-100x objectives, a Prior ProScan II stage, and an Olympus DP74 CMOS camera; images were analyzed using CellSens software.

### Cytokine microarray

After cardiac puncture, 100–150 μL of whole blood were collected into BD Microtainer serum separator tubes (gold cap), allowed to clot for at least 30 minutes at RT. Serum was separated by centrifugation at 10,000 × *g* and 4°C for 10 minutes and stored at −80 ° C until needed. For multiplex cytokine analysis, serum was assayed for the following murine cytokine levels by ELISA (Boster Biological Technology, CA). Briefly, forty-four cytokines and chemokines were measured with two separate kits (Millipore-Sigma), a 32-plex (G-CSF, GM-CSF, IFNγ, IL-1α, IL-1β, IL-2, IL-3, IL-4, IL-5, IL-6, IL-7, IL-9, IL-10, IL-12(p40), IL-12(p70), IL-13, IL-15, IL-17, IP-10, KC, LIF, LIX, MCP-1, M-CSF, MIG, MIP-1α, MIP-1β, MIP-2, RANTES, TNFα, VEGF) and a 12-plex (6Ckine/Exodus2, Erythropoietin, Fractalkine, IFNβ−1, IL-11, IL-16, IL-20, MCP-5, MDC, MIP-3α, MIP-3β, and TARC), using the Luminex^™^ 200 xMAP technology. Raw and the extrapolated values obtained from standard curves generated for each cytokine and chemokine.

### Statistical analysis

Statistical analyses were performed using GraphPad Prism 10.5.0 software (La Jolla, CA). Values are represented as the mean ± SD for at least two or three independent experiments. Survival data were plotted using Kaplan-Meier curves, and the different treatment groups were compared using the Log-rank (Mantel-Cox) test. The two-tailed unpaired Student’s *t*-test was used to compare two independent groups, and the one-way ANOVA was used to compare three or more groups. *P* values are reported as follows: not significant (ns) *P* > 0.05, * *P* < 0.05, ** *P* < 0.01, *** *P* < 0.001, **** *P* < 0.0001. For cytokine analysis, we used a multiple linear regression model with log-transformed concentration and chose an increase of 2-fold over PBS as significant while a ratio under 0.8-fold was considered a decrease in protein expression.

## Supplementary Files

This is a list of supplementary files associated with this preprint. Click to download.

- CarmonaSupplementaryMaterials.docx
- CarmonaSupplementaryTables.xlsx

## Data Availability

The data that support the findings of this study are available in the article.
